# Dynamics of Jasmonate Metabolism upon Flowering and across Leaf Stress Responses in *Arabidopsis thaliana*

**DOI:** 10.3390/plants5010004

**Published:** 2016-01-06

**Authors:** Emilie Widemann, Ekaterina Smirnova, Yann Aubert, Laurence Miesch, Thierry Heitz

**Affiliations:** 1 Institut de Biologie Moléculaire des Plantes, CNRS-UPR2357, associée à l'Université de Strasbourg, 12 rue du Général Zimmer, 67084 Strasbourg Cedex, France; widemann@mail.tsinghua.edu.cn (E.W.); ekaterina.smirnova@ibmp-cnrs.unistra.fr (E.S.); ian.aubert@hotmail.fr (Y.A.); 2 Laboratoire de Chimie Organique Synthétique, Unité Mixte de Recherche 7177, Université de Strasbourg, 67008 Strasbourg Cedex, France; lmiesch@unistra.fr

**Keywords:** Arabidopsis, JA-Ile, cytochrome P450, CYP94, amidohydrolase, hormone homeostasis, jasmonate catabolism

## Abstract

The jasmonic acid (JA) signaling pathway plays important roles in adaptation of plants to environmental cues and in specific steps of their development, particularly in reproduction. Recent advances in metabolic studies have highlighted intricate mechanisms that govern enzymatic conversions within the jasmonate family. Here we analyzed jasmonate profile changes upon *Arabidopsis thaliana* flower development and investigated the contribution of catabolic pathways that were known to turnover the active hormonal compound jasmonoyl-isoleucine (JA-Ile) upon leaf stress. We report a rapid decline of JA-Ile upon flower opening, concomitant with the massive accumulation of its most oxidized catabolite, 12COOH-JA-Ile. Detailed genetic analysis identified CYP94C1 as the major player in this process. CYP94C1 is one out of three characterized cytochrome P450 enzymes that define an oxidative JA-Ile turnover pathway, besides a second, hydrolytic pathway represented by the amido-hydrolases IAR3 and ILL6. Expression studies combined with reporter gene analysis revealed the dominant expression of *CYP94C1* in mature anthers, consistent with the established role of JA signaling in male fertility. Significant *CYP94B1* expression was also evidenced in stamen filaments, but surprisingly, CYP94B1 deficiency was not associated with significant changes in JA profiles. Finally, we compared global flower JA profiles with those previously reported in leaves reacting to mechanical wounding or submitted to infection by the necrotrophic fungus *Botrytis cinerea*. These comparisons revealed distinct dynamics of JA accumulation and conversions in these three biological systems. Leaf injury boosts a strong and transient JA and JA-Ile accumulation that evolves rapidly into a profile dominated by ω-oxidized and/or Ile-conjugated derivatives. In contrast, *B. cinerea*-infected leaves contain mostly unconjugated jasmonates, about half of this content being ω-oxidized. Finally, developing flowers present an intermediate situation where young flower buds show detectable jasmonate oxidation (probably originating from stamen metabolism) which becomes exacerbated upon flower opening. Our data illustrate that in spite conserved enzymatic routes, the jasmonate metabolic grid shows considerable flexibility and dynamically equilibrates into specific blends in different physiological situations.

## 1. Introduction

Jasmonic acid (JA) and its derivatives, commonly referred to as jasmonates (JAs), are lipid-derived phytohormones with a plethora of functions. JAs play important roles in plant growth and development as well as in plant biotic and abiotic stress responses in cooperation with other plant hormones [1,2,3,4].

The details of JA biosynthesis in *A. thaliana* have been extensively described (e.g., in [5]). Briefly, JA synthesis starts in plastid membranes when phospholipases A_1_ like Deficient in Anther Dehiscence 1 (DAD1) or related enzymes releases α-linolenic acid (C18:3) [6,7], which then undergoes oxidation by a 13-lipoxygenase into 13-hydroperoxy-octadecatrienoic acid (13-HPOT). By the successive action of 13-allene oxide synthase (AOS) and allene oxide cyclase (AOC), 13-HPOT is converted to 12-oxo-phytodienoic acid (OPDA). OPDA is then transferred to peroxisomes, where its cyclopentenone ring is reduced by OPDA reductase 3 (OPR3) and its carboxylic side chain undergoes three rounds of beta-oxidation involving acyl-CoA oxidase (ACX1) to yield JA.

Upon stress or developmental signals, JA can be channeled through at least 6 different metabolic routes [4,8] thus generating a vast array of compounds, some of them being associated with distinct biological activities [9]. How jamonate fluxes and different compound pool sizes are controlled remains largely unknown. Part of the accumulated JA is conjugated to various amino acids via the action of JAR1 or related enzymes, and one major conjugate, jasmonoyl-isoleucine (JA-Ile), represents a bioactive form of the hormone [10,11]. JA-Ile specifically promotes the assembly of a co-receptor composed of COI1 (CORONATINE INSENSITIVE1), an F-box protein component of the E3 ubiquitin ligase SCF^COI1^, and transcriptional repressors of the JAZ (JASMONATE ZIM-DOMAIN) protein family. In the absence of JA-Ile, JAZs along with a number of associated co-repressors, bind to and block transcription factors (e.g., MYC2/3/4) [12] that target downstream genes involved in specific JA responses. The assembly of the COI1-(JA-Ile)-JAZ complex triggers the ubiquitination of JAZs proteins leading to their proteolytic degradation via the ubiquitin/26S proteasome pathway [11], ultimately releasing the transcription of JA-responsive genes from repression.

**Figure 1:**
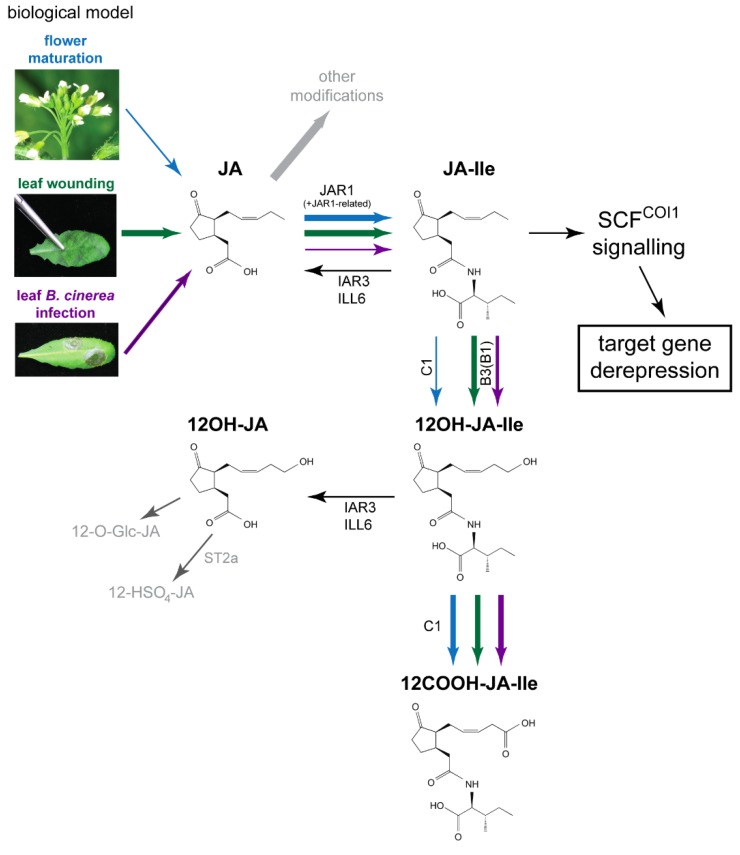
Proposed model for interconversion routes between jasmonic acid (JA) and its Ile-conjugated/ω-oxidized derivatives upon leaf stress responses or flower maturation, with emphasis on quantitative aspects as explained in main text. Upon leaf stress responses (wounding or *B. cinerea* infection) or flower maturation JA synthesis is activated to different extents, mechanical wounding providing the strongest burst. JA can be modified via different metabolic routes, one being the formation of jasmonoyl-isoleucine (JA-Ile) by JAR1 or related conjugating enzymes. JA-Ile is then either ω-oxidized by the action of CYP94 family enzymes to its hydroxy- (12OH-JA-Ile) or carboxy- (12COOH-JA-Ile) derivatives. In addition, JA-Ile and 12OH-JA-Ile can be hydrolyzed by the amidohydrolases IAR3 and ILL6, leading to the formation of JA and 12OH-JA, respectively. The thickness of arrows for the main JA metabolic pathway (from JA to 12COOH-JA-Ile) reflects the abundance of a given reaction product in each physiological context as detailed in Discussion. Comparative analysis of jasmonate profiles in the three biological systems as described in this paper shows that adequate JA-Ile levels can be achieved with distinct upstream and downstream metabolite pool sizes through the action of common enzymes. Blue—flower maturation, green—leaf wounding, purple—leaf *B. cinerea* infection. Predominant enzymes performing a given reaction are mentioned as follows: CYP94C1: C1, CYP94B1: B1, CYP94B3: B3, ST2a—sulfotransferase 2a. The compounds are shown in (3,7)-cis stereochemistry.

Different JAs derivatives have long been known to accumulate differentially in various plant organs or physiological situations. The formation of some of these derivatives was initially proposed to represent JA inactivation pathways [13]. Later, the identification of JA-Ile as the bioactive signal mediating most COI1-mediated JA responses has renewed interest for metabolic studies, particularly those addressing JA-Ile turnover mechanisms. As JA-Ile is a master switch orchestrating broad genetic reprogramming, and antagonizing other hormonal responses [14], its level need to be tightly controlled to provide flexible and reversible stress or developmental signaling. Two enzymatic pathways for JA-Ile removal have recently been identified and operate by enzymatic oxidation of the JA moiety (JA-Ile oxidative pathway) or by cleavage of the amide bond between JA and Ile (JA-Ile hydrolytic/deconjugation pathway), repectively. On the one hand, oxidation of JA side chain terminal carbon (hence the term ω-oxidation) is achieved by the action of cytochromes P450 of the CYP94 family, with CYP94B3 and CYP94C1 catalyzing preferential formation of 12OH-JA-Ile and 12COOH-JA-Ile, respectively. We and others have extensively analyzed the genetics and biochemistry of this oxidative pathway (Figure 1). CYP94 induction in part controls the duration and amplitude of the JA-Ile pulse upon leaf stress and consequently attenuates defense and resistance responses to herbivore and microbial attacks [15,16,17,18]. CYP94-mediated ω-oxidation of JA-Ile results in a strong decrease of its hormonal activity. Indeed, whereas 12OH-JA-Ile is still able to weakly promote the formation of COI1-JAZ complexes or to induce detectable transcriptional responses, 12COOH-JA-Ile is completely inactive [15,19]. CYP94 catalysis may be even more diversified as CYP94B1, B3 and C1 enzymes can also oxidize other JA conjugates including JA-valine or JA-phenylalanine [20,21], and CYP94C1 activity generates 12CHO-JA-Ile aldehyde *in planta* [21].

On the other hand, the JA-Ile deconjugation or hydrolytic pathway was first identified in the wild tobacco *Nicotiana attenuata* where *JASMONOYL-ISOLEUCINE HYDROLASE 1 (JIH1)* encodes an amidohydrolase (AH) whose silencing strongly enhanced JA-Ile levels and several JA-Ile-controlled direct and indirect defenses [22]. In *Arabidopsis*, *IAR3* and *ILL6*, two *JIH1* orthologous genes exhibit a strong co-regulation with the JA pathway. IAR3 was initially described as cleaving exogenously-fed auxin-amino acid conjugates, liberating active auxin *in planta* during seed germination [23]. However, in stressed leaves, JA conjugates are much more abundant than auxin conjugates, and genetic analysis indicated that induced IAR3 and ILL6 activity acts preferentially on JA-Ile and 12OH-JA-Ile substrates [24]. The identification of this latter reaction provided the first, indirect biosynthetic route towards tuberonic acid (12OH-JA) formation (Figure 1) and its sulfated and glucosylated derivatives. The oxidative and hydrolytic JA-Ile catabolic pathways thus work coordinately to attenuate JAs signaling [19] and as a consequence shape the dynamic JAs signatures in defending leaves [9,15,16].

Plant reproduction has been recognized as a developmental process where the JA pathway is crucial. Long-standing genetic evidence in *Arabidopsis* has shown that most mutants in JA synthesis (*fad378*, *dad1*, *aos*, *opr3* *acx1/5*) and perception (*coi1*) are male sterile [7,25,26,27,28,29,30], due to defects in late stage of *Arabidopsis* flower development. These genotypes are impaired in filament elongation, pollen maturation and finally anther dehiscence, thereby preventing well-timed pollination of the pistil [7,31,32]. Cumulative evidence has elucidated complex hormonal networks which orchestrate flower development and maturation [33,34]. Auxin-regulated events promote JA biosynthesis in stamens that in turn activates a complex of bHLH-MYB transcription factors (TFs) involving MYC2/3/4/5 [35] and MYB21/24. These latter are two JA-inducible TFs that are guarded by JAZ repressors [36] and that were initially identified as governing proper stamen development [37]. The general requirement of JA signaling in flower development and fertility was more recently found conserved in other plant species, but striking variations were evidenced. For example, in rice, defects along the metabolic or signaling pathway affect floret opening and anther dehiscence, but also spikelet development [38,39,40]. In maize, JA biosynthetic mutants show even earlier flower developmental defects including sex determination of male reproductive organs [41,42]. A different situation is known in tomato, where the *jai1* mutant that is defective for the homolog of the F-box protein COI1 is female sterile [43]. More recent analysis seems to involve OPDA as the specific signal required for proper embryo development [44]. Finally, in the wild tobacco *N. attenuata*, JA-Ile coordinates metabolic networks required for anthesis and floral attractant emission [45].

Consistent with this genetic evidence for important roles in plant reproduction, many JAs have been described as accumulating abundantly in flowers from different species [46]. Early investigations in tomato revealed a specific jasmonate blend in distinct flower organs [47]. All these data pre-dated the identification of JA-Ile as a major signal in this hormonal class or the still uncomplete unraveling of JA-Ile catabolic pathway. However, in contrast to their analysis in leaf stress responses, dynamics of JA-Ile metabolism during the crucial milestone that represents flower development have not been investigated in any plant species so far.

Here we present an investigation of JA-Ile and JAs dynamics in Arabidopsis flowers. Results demonstrate that CYP94C1-mediated JA-Ile oxidation occurs at Arabidopsis flower opening. We then compare these data with JA dynamics previously determined in leaves reacting to mechanical wounding or to infection by the fungus *Botrytis cinerea.* Despite being the site of active JA-Ile metabolism and signaling, these three biological models develop clearly distinct JA profiles, reflecting different limiting steps and fluxes through conjugation/deconjugation and ω-oxidation processes.

## 2. Results

### 2.1. Oxidation of JA-Ile Increases at Flower Opening

**Figure 2:**
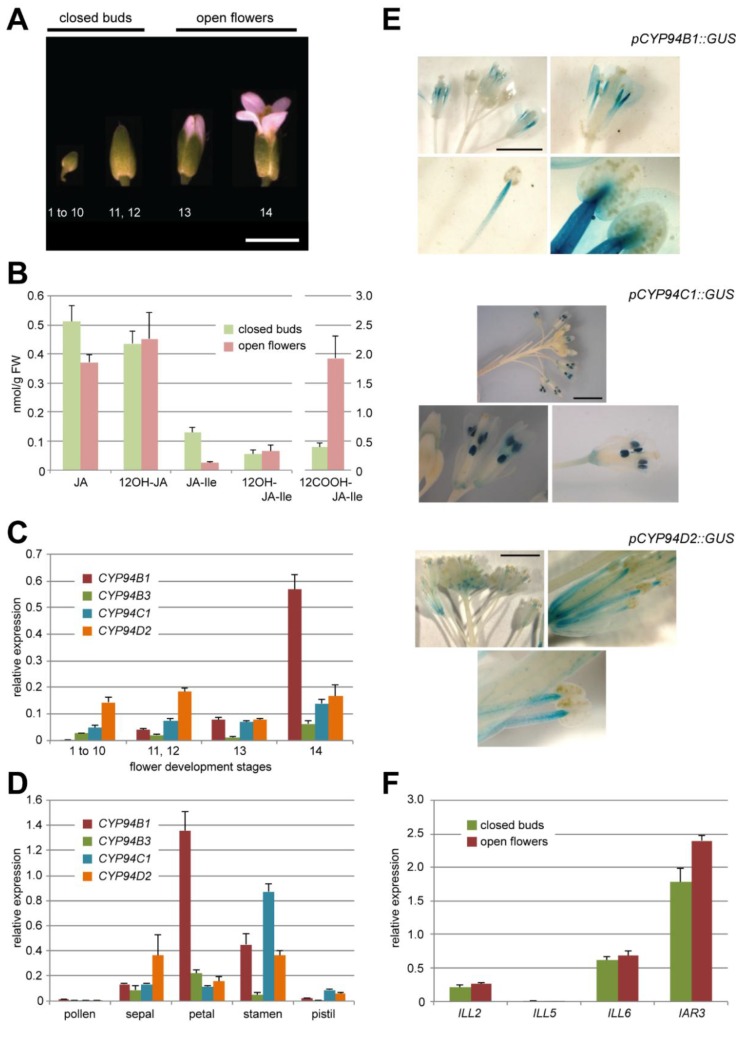
(**A**) *A. thaliana* flower development stages corresponding to that indicated in [32]. Stages 1–12 further referred to closed buds, stages 13 and 14—to open flowers (as indicated above). Scale bar corresponds to 3 mm. (**B**) UPLC-MS/MS quantification of five JAs content in *Col0* closed flower buds and open flowers. Note the different scale for 12COOH-JA-Ile on the right axis. Values are presented as means with SD of three independent biological repeats. (**C**, **D**) Relative expression levels of 4 *CYP94* genes *(–B1, –B3, –C1, –D2)* in *Col0* flowers distributed by development stages (C) or flower organs (stade 14)(D). Expression levels are normalized by expression of two reference genes. Values are presented as means with SD of three technical repeats. (**E**) Typical GUS staining pattern of flowers of *pCYP94C1::GUS*, *pCYP94B1::GUS* and *pCYP94D2::GUS* transgenic lines at different magnitude (from whole inflorescence to a separate anther). Two independent lines were analyzed for each construct. Scale bar corresponds to 3 mm. (**F**) Relative expression levels of 4 *AH* genes *(ILL2, ILL5, ILL6, IAR3)* in *Col0* closed flower buds or open flowers. Expression levels are normalized by expression of two reference genes. Values are presented as means with SD of three technical repeats.

*Arabidopsis* flowers were collected as two separate pools : closed flower buds corresponding to stages 1–12 defined by [32], and opening flowers of stages 13–14 (Figure 2A). Metabolites were extracted and samples analyzed quantitatively by UPLC-MS/MS for their contents in JA, 12OH-JA, JA-Ile and the two oxidized derivatives 12OH-JA-Ile and 12COOH-JA-Ile (Figure 2B). All compounds were readily detected both in closed buds and open flowers, the most abundant compounds being JA in the former and 12COOH-JA-Ile in the latter. 12OH-JA and 12OH-JA-Ile levels were stable upon flower opening. In contrast, drastically reduced JA-Ile quantities were recorded in open flowers, simultaneous with a large increase in 12COOH-JA-Ile abundance that reaches 2 nmol·g·FW^−1^. These temporal evolutions suggest that an active JA-Ile oxidation process to 12COOH-JA-Ile takes place at flower opening. The low and steady content in 12OH-JA-Ile may indicate that this intermediate substrate is rapidly turned over toward the more oxidized conjugate and/or cleaved to 12OH-JA by amidohydrolases.

### 2.2. JA-Ile Catabolic Genes Display Dynamic Expression upon Flower Development

#### 2.2.1. The JA-Ile Oxidase-Encoding Genes *CYP94B1*, *CYP94B3* and *CYP94C1* Are Differentially Expressed at Flower Opening

Expression of the six genes in the *CYP94* family was analyzed by real-time PCR in flowers collected at a higher temporal resolution, in 4 samples covering all stages of development from floral meristem up to open flowers (stage 1 to 10, 11–12, 13, 14). *CYP94D1* yielded no signal and *CYP94B2* expression was very weak. Consequently, these genes were not further studied. The expression of the other 4 genes (*CYP94B1*, *B3*, *C1* and *D2*) was reliably detected in all samples. *CYP94D2* is a gene of unknown function which does not encode a JA-Ile oxidase; its global expression fluctuated within a two-fold range through the different stages of developing flowers. Interestingly, the expression of *CYP94B1*, –*B3* and –*C1*, all known as encoding JA-Ile oxidases acting in stressed leaves [15,16,17,18,19] peaked at stage 14 open flowers (Figure 2C), concomitant with JA-Ile oxidation (Figure 2B). *CYP94B3* was the weakest expressed gene, whereas *CYP94B1* expression was most prominent. When flower organs from stage 14 were dissected and analyzed, *CYP94B1* expression was highest in petals and stamens (Figure 2D) whereas *CYP94C1* expression appeared largely stamen-specific. All four genes were very weakly expressed in pollen. Finally, promoter-reporter transformants were generated for *CYP94B1*, *C1* and *D2* and analyzed for β-glucuronidase (GUS) expression in inflorescences. Representative patterns are shown in Figure 2E. *pCYP94B1-GUS* displayed a diffuse staining in distal part of petals and a gradually more dense signal in the upper part of elongating stamen filaments, confirming earlier observations by [19]. In contrast, *pCYP94C1-GUS* marked exclusively and strongly anthers in open flowers at stages 13–14. *pCYP94D2-GUS* stained young flower bud stalks and later vascular tissues in elongated anther filaments of open flowers. These data reveal organ-specific expression patterns that are consistent with qPCR results and point to a possible role of some *CYP94* genes in JA-Ile turnover in *Arabidopsis* flowers.

#### 2.2.2. Amidohydrolases Are Expressed at the Same Level in Closed Buds and Open Flowers

As IAR3 and ILL6, two members of the AH enzyme family, act in JA-Ile turnover in concert with CYP94 enzymes upon leaf wounding [19,24], we investigated their global expression profiles by RT-qPCR in closed and open *Col0* flowers (Figure 2F). Among the 7 members of the AH family we also analyzed *ILL5* as a third specimen of JA-coregulated gene, and *ILL2* for which no link with the JA pathway is known. For *ILL5* no expression was found at either stage. For the other 3 analyzed genes, expression levels were largely unchanged upon floral development. *ILL2* displayed low expression, while *IAR3* exceeded *ILL6* expression and was slightly increased at flower opening. These results are consistent with *AH* gene expression reported previously in *Arabidopsis* inflorescences [23].

### 2.3. CYP94C1 Is the Major Enzyme for JA-Ile Oxidation upon Arabidopsis Flower Opening

**Figure 3:**
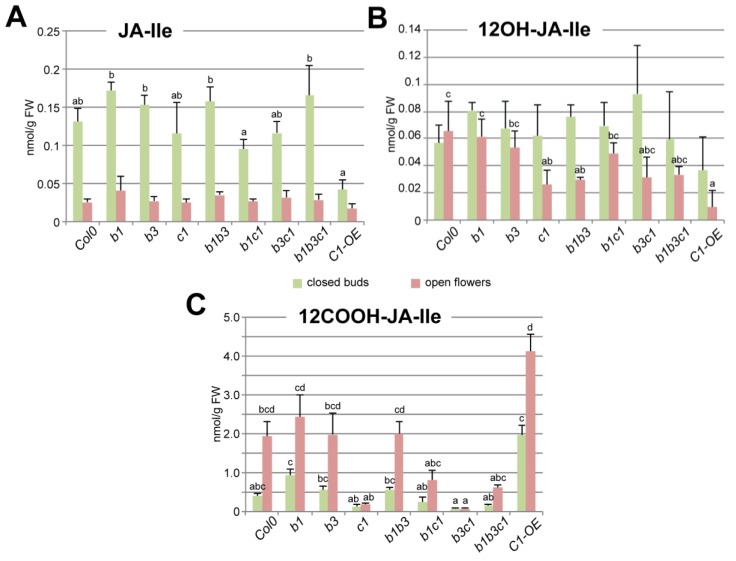
UPLC-MS/MS analysis of conjugated JAs (JA-Ile, 12OH-JA-Ile and 12COOH-JA-Ile) abundance in closed buds or open flowers of *Col0* control plants and of simple mutants *cyp94b1, –b3 and –c1*, double mutants *cyp94b1b3, –b1c1 and –b3c1*, triple mutant *cyp94b1b3c1*, and *CYP94C1* over-expressor mutant *(C1-OE).* Values are presented as means with SD of three independent biological repeats. Statistical analysis was applied separately to the pool of data corresponding to either closed buds or open flowers. Columns are labelled with different letters indicating a significant difference in given jasmonate content between genotypes as determined by Kruskal-Wallis one-way analysis of variance (ANOVA) (*p* < 0.05). Absence of letters corresponds to the absence of significant difference in a given JA abundance in different analyzed genotypes.

As increased expression of some *CYP94* genes at flower opening—particulary in stamens—correlated with active JA-Ile oxidation, we determined the impact of CYP94 deficiency on unoxidized or oxidized JA-Ile levels. Metabolic extracts of closed flower buds (stages 1–12) or opening flowers (stages 13–14) from single, double, and triple mutants in *CYP94B1*, –*B3* and –*C1* genes and also p35S-*CYP94C1* overexpressing plants (hereafter *C1-OE*) were submitted to JA-Ile profiling by UPLC-MS/MS analysis. Surprisingly, all loss-of-function mutants displayed near-WT JA-Ile levels at the two developmental stages analyzed. In contrast, the *C1-OE* line showed a significant (70%) decrease of JA-Ile content in closed buds but barely reduced in open flowers compared to WT (Figure 3A). 12OH-JA-Ile, the initial JA-Ile oxidation product, was accumulated to low levels, which were further depressed to variable extents in the different mutants, notably in *cyp94c1* and *cyp94b1b3*. This indicates that all three *CYP94* genes act redundantly in the formation and build-up of this intermediate. Furthermore, its levels are more strongly reduced in *C1-OE* open flowers, indicating a higher consumption. Finally, the levels of 12COOH-JA-Ile, the end product of CYP94-dependent JA-Ile oxidation pathway were reduced to various entents in open flowers of CYP94C1-deficient lines (*c1*, *b1c1*, *b3c1* and *b1b3c1*) (Figure 3C). Reciprocally, ectopic CYP94C1 overexpression enhanced 12COOH-JA-Ile abundance in closed buds as well as in open flowers compared to WT. These results indicate that, as in fungus-infected leaves [15], CYP94C1 is the major enzyme for oxidative JA-Ile catabolism upon flower maturation. Unexpectedly, when the *cyp94c1* mutation was combined with *cyp94b1* (in *b1c1* and *b1b3c1*), 12COOH-JA-Ile levels were higher in open flowers than in lines where CYP94B1 was functional (in *c1* and *b3c1*). This observation indicates the possibility of an alternative route that may substitute to CYP94 deficiency to make 12COOH-JA-Ile [48].

Of note, no obvious flower developmental defects were recorded for any of the CYP94-modified lines, in contrast to CYP94B1-OE and CYP94B3-OE lines reported previously as male sterile in [18,19]. Very high expression in these latter lines may deplete JA-Ile below a threshold level required for proper flower development.

## 3. Discussion

### 3.1. CYP94C1 Is a Major Player in JA-Ile Oxidation upon Floral Opening

JA-Ile inactivation mechanisms have been recently characterized, mostly in leaf stress responses where *CYP94* and amidohydrolase (*AH*) genes are induced together with JA biosynthetic and signaling genes [15,16,17,18,19,24]. These catabolic pathways inactivate the hormone by ω-oxidation or conjugate cleavage and thereby attenuate defense responses. They also largely remodel jasmonate metabolic profiles (or jasmonate signatures) in attacked tissues. The precise implications of the accumulation of various JAs deriving from JA-Ile breakdown remain to be determined. In wounded leaves rapid JA-Ile inactivation/removal is achieved by the concerted action of CYP94 and AH enzymes. Under this stimulus, CYP94B3 and CYP94C1 work sequentially: CYP94B3 contributes predominantly to the initial oxidation to 12OH-JA-Ile and CYP94C1 accounts for the conversion of this intermediate to 12COOH-JA-Ile [16]. AH not only deconjugates JA-Ile but also acts on 12OH-JA-Ile to generate 12OH-JA [24]. In contrast, in leaves reacting to fungal infection by *Botrytis cinerea*, CYP94C1 was the predominant member controlling hormone oxidation [15]. The genetic and biochemical dissection of these two biological models has defined a complex jasmonate metabolic grid where JA-Ile acts as a metabolic hub directing the synthesis of numerous conjugated and unconjugated derivatives [9].

Here we used the same genetic and analytical tools to investigate JA-Ile turnover in *Arabidopsis* floral development. We show that at flower opening, a rapid decrease in JA-Ile levels matches the massive accumulation of 12COOH-JA-Ile. These results corroborate similar profiles obtained in the frame of the study of CYP715A1 with the methods we developed here [48]. The present genetic analysis of the CYP94 family revealed that CYP94C1 alone accounts for this conversion, with a comparatively low pool size of the intermediate 12OH-JA-Ile. Ectopic CYP94C1 overexpression had the most (relative to WT) impact in closed flower buds, when native CYP94C1 expression is still low. In contrast to the situation in wounded leaves, we did not detect a significant contribution of CYP94B3 to JA-Ile homeostasis in flowers. Remarkably, no significant increase of JA-Ile level was observed in any analyzed mutated line relative to WT even in the double *cyp94b3c1* mutant where 12COOH-JA-Ile formation was virtually abolished. Additionally, the drop in JA-Ile levels in open flowers was similar to WT in all lines. This indicates that in contrast to injured leaves where the CYP94 pathway is essential for rapid clearance of JA-Ile [16], flowers are able to eliminate the hormone through other means. Compensation of CYP94-deficiency by a higher expression of the AH pathway would be a possibility, but *IAR3* and *ILL6* transcript levels were not found significantly perturbed in flowers of CYP94-deficient plants (data not shown).

The impact of the different mutations on global flower JA-Ile homeostasis is consistent with known enzymatic characteristics of the three CYP94 proteins [16] and with their expression profiles. The use of *CYP94* promoter-reporter lines provided novel insights into possible sites of JA-Ile turnover. *pCYP94B1::GUS* reporter labelled elongated filaments and petals, in accordance with JA being synthesized in young filaments [7,49] to promote their elongation and anther dehiscence. *CYP94B1* expression may therefore initiate signal deactivation during filament elongation. However, as no metabolic alteration in JA-Ile turnover was evidenced in total flower extracts of CYP94B1-deficient plants, identifying the exact substrate of CYP94B1 will require analysis in isolated organs. The specific expression of *CYP94C1* in dehisced anthers is consistent with a termination of JA-Ile signaling that is active in this organ. From genetic data we infer that most 12COOH-JA-Ile measured in stage 14 flowers originates from JA-Ile and 12OH-JA-Ile oxidation in anthers. These expression patterns suggest a sophisticated organization of JA-Ile control, where an increasing oxidation gradient from the base of filament toward the anther may be coupled with the transport of intermediate forms. Consistent with unchanged JA-Ile levels, CYP-deficient lines did not display altered expression of target genes, including *JAZ*, *MYB21/24* or the defensive myrosinases *TGG* (data not shown). The molecular function of active JA-Ile catabolism at late stamen development is thus not clear. A future challenge will be to map the sites of accumulation and conversion of JA/JA-Ile metabolites in *Arabidopsis* flowers to understand JA dynamics in reproductive development.

### 3.2. Comparative Analysis of JA Pools in Flower Development and Leaf Stress Responses

JA signaling plays important roles in many aspects of plant life. We established that the master regulator JA-Ile is at the crossroads of a complex metabolic grid whose dynamics are highly controlled to fine-tune flexible stress or developmental responses (Figure 1). In recent years we developed genetic and analytical tools to investigate the metabolic fate of JA-Ile in three distinct models, flower development, as described above, and two types of leaf stress responses reported previously: mechanical wounding [16,21,24] and infection by the necrotrophic fungus *Botrytis cinerea* [15], which reflect processes activated upon plant-insect and plant-microbe interactions, respectively.

In flowers, endogenous signals involving sequential hormonal cascades upregulate JA synthesis which then activates coordinated developmental processes that end up in the timely release of fertile pollen [50]. Upon leaf biotic stress, defense responses can be induced by two distinct and antagonistic branches of the JA/JA-Ile pathway [51]. On one hand, the attack by herbivorous insects, which can be partially mimicked by mechanical wounding, provokes a massive synthesis of JAs [52] where JA-Ile signals synergistically with abscisic acid (ABA) and de-represses MYC2-dependent transcription of defense genes [53,54]. On the other hand, resistance to necrotrophic microbes relies on the concomitant activation of JA and ethylene signals that are integrated by the AP2/ERF/ORA59 transcription factors. In these three biological contexts, the JA metabolic pathway receives input from different upstream activation factors, which translates into particular JA profiles/dynamics, and ultimately regulates distinct sets of target genes.

**Figure 4:**
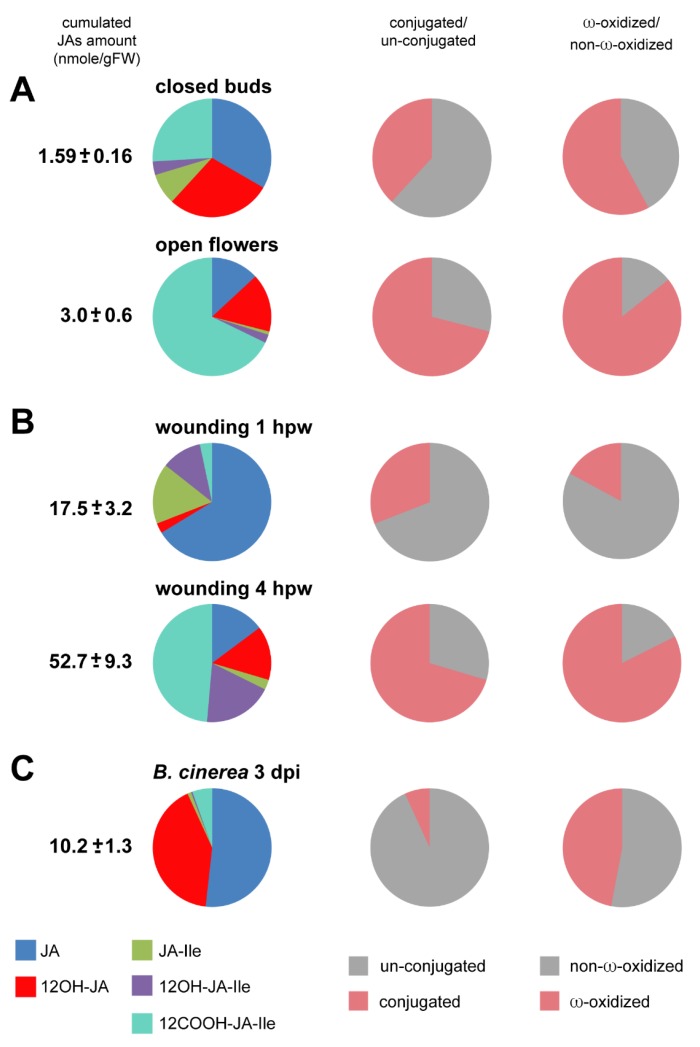
Pie diagrams of five JAs content in three biological systems**:** In left column, the number indicates cumulated amounts (nmol·g·FW^−1^) of JA, 12OH-JA, JA-Ile, 12OH-JA-Ile and 12COOH-JA-Ile as means with SD of three independent biological repeats. The left column pies indicate the proportions of conjugated (JA-Ile + 12OH-JA-Ile + 12COOH-JA-Ile) *versus* un-conjugated (JA + 12OH-JA). The right column pies indicate the proportions of non-ω-oxidized (JA + JA-Ile) *versus* ω-oxidized (12OH-JA + 12OH-JA-Ile + 12COOH-JA-Ile) JAs. (**A**) flower maturation: closed buds or open flowers (**B**) leaf wounding: 1 h post-wounding (hpw) or 4 hpw (**C**) leaf *B. cinerea* infection: 3 days post-inoculation (dpi).

In the next section, we comment on quantitative and qualitative differences in JA homeostasis in these different models. We focused on the temporal distribution of a set of 5 relevant JAs across these biological systems, although the complement of JA derivatives undoubtedly extends to many more compounds. A synthetic view of the compared data is represented in Figure 4 in the form of pie diagrams. In the first column the cumulated amount of these compounds (JA, 12OH-JA, JA-Ile, 12OH-JA-Ile and 12COOH-JA-Ile) is given along with their relative distribution (in percentage). The middle and right column depict respectively the amino acid-conjugation and ω-oxidation status of the JA blend monitored in each analysis.

#### 3.2.1. JA Profile Evolution upon Flower Opening

Among the material we examined, the flower samples displayed the lowest content in JAs, with a total around 3 nmol·g·FW^−1^. This result has likely two origins: the collection method combined in one sample all stages of closed flower buds (stages 1–12), including early stages where JA synthesis may not have started. In addition, extracting JAs from total (undissected) buds as performed in this study did not give access to organ-specific signal that was likely diluted by neighboring tissues that are poor in JAs. As a net result, JA, 12OH-JA and 12COOH-JA-Ile were found equally abundant in closed buds, followed by JA-Ile, illustrating that major steps in JA metabolism are operating before anthesis (Figure 4A). Upon floral opening, two major changes are detected in WT flowers: a 5-fold reduction in JA-Ile and an approximately 4-fold increase of 12COOH-JA-Ile. As a consequence, in open flowers, there is a tremendous shift from JA-Ile towards a large pool of 12COOH-JA-Ile, the ratio between these two compounds reaching about 1 to 75 (Figure 4).

#### 3.2.2. JA Profile Evolution upon Mechanical Wounding

Mechanical wounding is a convenient laboratory model to trigger massive transcriptional responses reminiscent of those activated by an herbivore insect attack. It provides a severe and synchronous stimulus that rapidly activates JA synthesis, signaling, and catabolism [9]. Hence, the cumulated amounts of the 6 analyzed JAs reach about 50 nmol·g·FW^−1^ at 4 h post-wounding (hpw).

In this material, stimulated cells undergo waves of molecular events that can be distinguished over time. Here we used results previously published in [16] and [24] and extracted data at the 1^st^ and 4^th^ hpw that correspond to two different stages of the JA metabolic response (Figure 4B). At 1 hpw JA-Ile is at its peak level [16], JA is the most abundant of the 5 compounds while 12OH-JA and 12OH-JA-Ile are still minor oxidized JAs. Overall, at this early time point, JAs are mainly unconjugated and unoxidized. At 4 hpw, a strong shift toward ω-oxidized and conjugated derivatives accumulation has occurred, 12COOH-JA-Ile representing nearly half of all compounds. This is reflective of the coordinated activation of conjugation/deconjugation and ω-oxidation pathways [16,24] that channel main fluxes toward the accumulation of downstream JAs while depleting the active hormone JA-Ile. The 4 hpw blend resembles qualitatively the distribution in open flowers, and is characteristic of the JA-Ile signal extinction phase.

#### 3.2.3. JA Profile in *B. cinerea*-Infected Leaves

Here the data are based on our previous investigation of JA profiles in response to *Botrytis* infection and the impact of CYP94 expression as described in [15]. Upon fungal infection, the dynamics of leaf stimulation are different from wounding as disease lesions develop radially starting from a single inoculation site on each half leaf. In this case, new tissues are stimulated continuously, therefore asynchronously, making this system less suitable to dissect the dynamics of metabolic conversions, sampled leaves combining both early and late metabolic events. Consequently, JA accumulation merely evolves quantitatively with lesion size, with little change in species distribution over time. A snapshot at 3 dpi of the JA profile in fungus-infected leaves is given in Figure 4C. The total 5-JA pool culminates here at about 10 nmol·g·FW^−1^ in leaves bearing two infection sites. Here, JA and 12OH-JA represent together >90% of all analyzed JAs. Oxidation is less prevalent compared to flowers or wounded leaves. Another striking feature of this profile is that unconjugated JAs dominate upon *B. cinerea* infection, and JA-amino acid conjugates and their derivatives remain minor in proportion. A possibility is that conjugating activity is low, or it may be reversed by high amido-hydrolase activity, for example IAR3 and ILL6 that generate JA and 12OH-JA [24]. Consequently, a large part of JA remains unmodified in reaction to *B. cinerea* infection.

## 4. Experimental Section

### 4.1. Plant Material and Growth Conditions

All *Arabidopsis* genotypes used in this paper were in *Col0* ecotype and were grown under a 12 h light/12 h dark photoperiod in a growth chamber (21/18 °C, respectively). T-DNA insertion lines were obtained from NASC center: *cyp94b1-1* (SALK_129672), *cyp94b3-1* (CS302217), *cyp94c1-1* (SALK_55455). Double and triple mutants were obtained by crossing of corresponding original homozygous lines with subsequent analysis of T2 generation [15]. CYP94C1 overexpressing line (C1-OE) was described in [16].

### 4.2. Jasmonate Profiles

Extraction of jasmonates and their quantification by UPLC-MS/MS were performed as described in [24].

### 4.3. RNA Extraction and Real-Time PCR

RNA extraction, reverse transcription and Reverse Transcriptase-qPCR analysis were performed as described in [16] using about 80 mg of whole flower material and 30 mg for samples of dissected flowers.

### 4.4. Histochemical GUS Staining

*pCYP94C1::GUS* lines were provided by Yves Millet (Massachussets General Hospital, Boston, MA, USA). *pCYP94B1::GUS* and *pCYP94D2::GUS* constructs were obtained by cloning of promoter sequences ranging from −1587 to −55 bp and from −1871 to −15 bp of corresponding genes, respectively (the A in ATG start codon is refered as +1), in pBGWFS7 vector with subsequent transformation of *Col0* plants. All material used for staining was from homozygous plants. GUS coloration was performed as described in [55] and samples were observed with a Leica Z16 APOA macroscope.

### 4.5. Statistical Analysis

Statistical analysis was performed using Infostat software (http://www.infostat.com.ar, Universidad Nacional de Córdoba, Argentina). Comparisons of sample medians were performed by Kruskal-Wallis one-way analysis of variance (ANOVA) (*p* < 0.05) to determine whether sample means were significantly different.

## 5. Conclusions

The analysis of JA-Ile turnover in Arabidopsis flowers further extends the involvement of CYP94 and amidohydrolase enzymes in the control of hormone turnover to a developmental case of JA signaling. Hormone analysis revealed highly active JA-Ile catabolism in maturing stamens, in accordance with extensive genetic data underlying the importance of jasmonates in male fertility in *Arabidopsis*. CYP94C1 was identified as the predominant enzyme for JA-Ile oxidative turnover, but surprisingly, the three studied CYP94 JA-Ile oxidases are dispensable for the rapid removal of JA-Ile upon flower anthesis, despite the large metabolic flux proceeding through JA-Ile oxidation. These observations open the possibility of alternative JA-Ile clearance route(s) in flowers. Based on the characterization of some enzymatic actors and likely sites of JA-Ile turnover, one of the next challenges will be to visualize specific JAs in flower organs and to elucidate how JA metabolism affects hormonal functions in plant reproduction.

The comparison of the dynamics of JA metabolism in flowers, a developmental model, with two previously characterized models of induced leaf defense, illustrates the great metabolic plasticity in the JA pathway. Even though JA-Ile is a central signal in all three systems, and common catabolic pathways are activated in the three situations, pools of individual JAs are very different, reflecting distinct bottlenecks in each physiological context. These specific features are highlighted in the pathway depicted in Figure 1, with variable arrow thicknesses reflecting presumed flux through each reaction. Additional parameters, such as interactions with other hormones and yet unknown routes in JA-Ile homeostasis may be crucial. Their search, together with new regulatory functions associated with JA metabolites will be the topic of new exciting investigations.
